# SGAtools: one-stop analysis and visualization of array-based genetic interaction screens

**DOI:** 10.1093/nar/gkt400

**Published:** 2013-05-15

**Authors:** Omar Wagih, Matej Usaj, Anastasia Baryshnikova, Benjamin VanderSluis, Elena Kuzmin, Michael Costanzo, Chad L. Myers, Brenda J. Andrews, Charles M. Boone, Leopold Parts

**Affiliations:** ^1^Donnelly Centre for Cellular and Biomolecular Research, University of Toronto, Toronto, M5S 3E1, Canada, ^2^Department of Molecular Genetics, University of Toronto, Toronto, M5S 1A8, Canada and ^3^Department of Computer Science and Engineering, University of Minnesota–Twin Cities, Minneapolis, MN, USA

## Abstract

Screening genome-wide sets of mutants for fitness defects provides a simple but powerful approach for exploring gene function, mapping genetic networks and probing mechanisms of drug action. For yeast and other microorganisms with global mutant collections, genetic or chemical-genetic interactions can be effectively quantified by growing an ordered array of strains on agar plates as individual colonies, and then scoring the colony size changes in response to a genetic or environmental perturbation. To do so, requires efficient tools for the extraction and analysis of quantitative data. Here, we describe SGAtools (http://sgatools.ccbr.utoronto.ca), a web-based analysis system for designer genetic screens. SGAtools outlines a series of guided steps that allow the user to quantify colony sizes from images of agar plates, correct for systematic biases in the observations and calculate a fitness score relative to a control experiment. The data can also be visualized online to explore the colony sizes on individual plates, view the distribution of resulting scores, highlight genes with the strongest signal and perform Gene Ontology enrichment analysis.

## INTRODUCTION

Screening for genetic or chemical-genetic interactions, which occur when a genetic or chemical perturbation leads to an extreme phenotype, provides a simple but powerful approach for revealing gene function and discovering the mode of action of bioactive molecules (1,2). In yeast, genome-scale reagents, such as the set of ∼5000 viable deletion mutants (3), collections of conditional temperature-sensitive mutants (4,5) and gene overexpression libraries (6,7), represent powerful resources for these screens. High-throughput methods for manipulating such arrays in yeast and other microorganisms have enabled automation of genetics in these model systems and often use colony size as a proxy for cell growth to infer a genetic interaction (1,8,9). The raw data can be a detailed movie, from which a growth curve is derived (10–12), but the most basic format is as a static plate image, which, somewhat surprisingly, is incredibly rich in functional information. However, individual colonies on plates are subject to positional effects, and thus, plate images require further processing and statistical analyses to extract meaningful biological insights (13).

Projects aimed at large-scale screening for genetic interactions have identified several sources of experimental error, which led to the development of analysis methods that are tailored to the resulting large data sets. For example, we have successfully applied Synthetic Genetic Array (SGA) (14) analysis to ascertain the effects of ∼5 million double gene deletions in yeast (1). A number of the best practices emerging from analysis of these data cannot be implemented directly for smaller screens, as they rely on a large assembly of controls and estimating biases from hundreds of different experiments to correct for systematic experimental errors. Moreover, other available methods are shared as code only (15–17), which may rely on obtaining expensive software licenses, a somewhat steep learning curve to compile and use the published code, or do not include all the analysis steps in a single web-based interface, making them less accessible. Consequently, laboratories that are interested in performing and analyzing a relatively small number of customized screens, such as those designed for detailed analysis of specific pathways, are lacking user-friendly data analysis solutions. Here, we present SGAtools, a simple complete solution for analyzing static image-based screens of ordered arrays of microbial cultures. SGAtools is an easy-to-use website that implements state-of-the-art normalization and quantification methods and includes some basic tools useful for visualizing the data.

## SGATOOLS WEB SERVER

**Figure 1.**
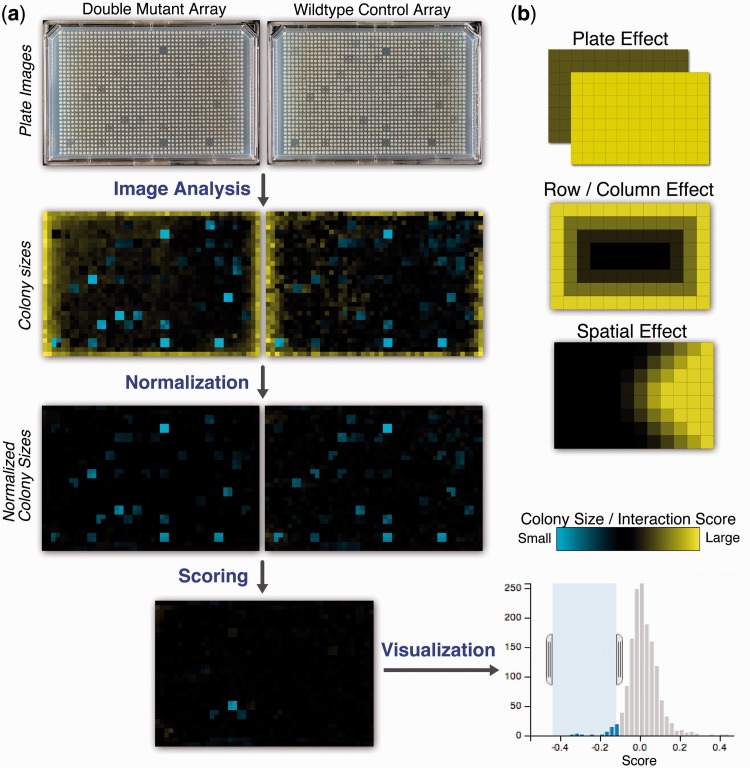
The SGAtools analysis pipeline. (**a**) Plate images are analyzed to produce raw colony size measurements, which are then normalized and scored. The scores can further be visualized and selected genes tested for enrichment in GO terms. Heatmap and histogram images of the steps are examples of the data analysis page output. (**b**) Three important biases in colony size measurements are corrected in SGAtools: (i) The plate effect accounts for differences in average colony size between plates; (ii) the row/column effect corrects for the outside colonies having more access to nutrients; and (iii) the spatial effect takes care of uneven thickness of medium in the plate.

SGAtools is a publicly available web server for common analysis tasks associated with low- to medium-throughput genetic screens. It comprises three independent tools that are combined into one pipeline (Figure 1a). First, raw digital images from the screen are processed to produce colony size measurements in a simple tab-delimited format. Second, the colony sizes are normalized to account for common biases that affect growth (13) and optionally scored to produce a measure of genetic interaction strength. Finally, the data and scores can be visualized interactively and tested for GO term enrichment (18). Results of the analyses can be downloaded after any of the steps.

>

## IMAGE ANALYSIS

The primary, and perhaps the most challenging, task associated with genetic screen analysis is quantifying colony sizes based on images taken of solid agar plates (10,11,19). We incorporated a modified version of the HT colony grid analyzer (20) in SGAtools. In brief, we rotate the plate image to make the rows of colonies horizontal, fit a grid based on the pixel intensities in the color channel with the highest contrast to find the colonies and count the foreground pixels at every grid node to produce colony size measures.

The user input to SGAtools image analysis is a set of plate image files of sufficiently high quality (160 dpi). In addition, the plate format used must be supplied to fit the right grid. Currently, pinned rectangular plates of 96 colonies (8 rows × 12 columns), 384 colonies (16 × 24), 768 colonies (two interleaved 384 colony configurations) and 1536 colonies (32 × 48) are supported. More advanced options allow choosing the image-cropping method to distinguish the plate edges from the agar area. We have found that the default choice of automated cropping works well for the vast majority of images, but the alternatives can help for special cases. A few failure modes for correctly segmenting the colonies remain and are documented on the website.

The selected images are uploaded and can then be analyzed. This process takes 6–10 s per image, depending on image size and resolution. After processing the images, the user has the option of downloading the output files and visually validating the image analysis results. One tab-delimited output file is created for each plate image, and every row of such a file gives the column, row, size and circularity of one colony. Selected plates can then be advanced to the normalization and scoring step.

SGAtools has pre-loaded yeast genetic array definitions commonly used to date, including the haploid viable deletion array (3), a gene overexpression array (6) and an array of strains harboring temperature-sensitive alleles of essential genes (4). A specific file name assigned to each plate image is used to link the images to a given array, and a screen plate to its corresponding control. The filename should have five underscore-delimited fields that correspond to (i) any prefix, (ii) plate type (‘ctrl’ or ‘wt’ for control plate, ‘dm’ for double mutant or other non-control), (iii) query strain name, (iv) plate number in the array, and (v) any suffix. For example, file ‘2013-05-12_ctrl_YML032C_2_rep3.jpg’ will be interpreted as the control for crossing the YML032C query strain into second plate of the array. The array itself can be specified at the next step.

## NORMALIZATION AND SCORING

Colony size measurements derived from screens using ordered mutant arrays are susceptible to systematic variation caused by several experimental factors. These factors include subtle differences in growth conditions, such as duration of incubation, from one array plate to the next, as well as factors that influence local nutrient availability and affect growth of different subsets of colonies on the same plate. These include location of the colony on the plate, gradients in growth medium volume caused by uneven preparation surfaces and neighboring mutant-strain fitness. Thus, to accurately quantify colony fitness, all of these factors, as well as specific batch effects, must be corrected. The normalization step implements the previously established methods (13) that are applicable for small-scale screens for this purpose. Below, we summarize them for completeness and highlight the differences to standard SGA screening analyses.

SGAtools corrects for three major sources of bias (Figure 1b). First, all plates are normalized to have the same median colony size. This assumes that most colonies have wild-type fitness (i.e. effects of the applied perturbation are rare) and removes any inconsistencies between plates that can be due to variable image size, timing of imaging, plate thickness and so forth. Second, colonies in each row and column are rescaled to have directly comparable size, as described in (13). This normalization is especially important for colonies toward the edge of the plate, which have more access to nutrients compared with the central ones, enabling them to grow to a larger size. Finally, the spatial correction accounts for correlations in colony sizes in a region of the plate. This effect is due to variable plate thickness and can be corrected by spatial smoothing of the colony sizes [see (13)]. Competition effect, another source of bias that can be accurately accounted for in large-scale screens (13), is implemented as a filter only (see later in the text).

In addition to correcting for broad plate effects, SGAtools implements filters to flag specific colonies that have unexpected sizes. The jackknife filter removes colonies that are different from its technical replicates. The linkage filter removes colonies where construction of the double deletion mutant requires a meiotic crossover between two linked genes, with the distance threshold determined by the user in kilobases. As a crossover between genetically linked genes is a relatively rare event, the associated colonies will be small because fewer double mutants are generated (21,22), and thus their sizes may not accurately reflect the double mutant fitness. Very large colonies are also removed, as they can indicate a contamination problem, or perhaps a result from genetically altered diploids that somehow escape the SGA selection system for growth of only haploid double mutant meiotic progeny. Finally, colonies that show a positive genetic interaction score (i.e. those that are larger than expected), but are located next to colonies with a negative interaction (i.e. those that are smaller than expected), are flagged as potential false positive interactions, as they have access to more nutrients owing to the small size of their neighbors (also known as the competition effect).

After normalization, the user can opt to score the screens against controls by quantifying the deviation from the expected fitness. Negative scores are generated when the double mutant displays a fitness defect that is more extreme than expected for the combined effect of the two single mutations, with the most extreme example being synthetic lethality. Positive interaction scores are generated when the double mutant displays a healthier phenotype than expected for the combined effect of the two single mutations, such as genetic suppression (13,23). This is done using the standard multiplicative score (*W_ij_−W_i_W_j_*), where *W_ij_*, *W_i_* and *W_j_* represent the fitness of the observed value in the experiment, the median fitness of all strains in the experiment (estimated from the control plate) and the median fitness of the array strain (estimated as the median value of all its replicates), respectively. The fitness values correspond roughly to the normalized colony size that is rescaled to have an average value of 1, and the scores quantify the deviation from the expected fitness. For reference, with our yeast SGA system, we find that multiplicative scores below −0.3 indicate a strong effect (e.g. synthetic lethal interaction) and should be visible in plate images by eye. In low-throughput screens, multiplicative scores between −0.1 and 0.1 are unlikely to correspond to reproducible effects, and positive scores between 0.1 and 0.3 should be treated with caution owing to the competition effect described earlier in the text. Similar to all genomic technologies, SGA screens can also give rise to false negative and false positive interactions. Their rates are affected by experimental conditions and human error and can vary from screen to screen, and, as a result, interactions identified using SGAtools should be independently confirmed.

SGAtools performs the normalization and scoring on a tab-delimited file containing the quantified colony sizes. The files can be uploaded by the user or automatically moved through from the image analysis step. Each tab-delimited input file must have row, column and colony size in the first three columns; the rest of the columns are ignored. To link colonies to genes, the user can select which array they were using or upload their own description. The files must be named according to the standard described in the image analysis section to use this feature. To score the data correctly, the technical replicate structure of the plate must be supplied. Currently, a single replicate, and four replicates in a 2 × 2 grid are supported. Multiple biological replicates of the same strain can be used as well by defining the same strain multiple times in the plate layout file. The output of normalization and scoring is a nine column tab-delimited file, which includes the raw colony size, normalized colony size, scores if computed and information about any filtering results (Table 1). A single file combining all results across plates, and averaging the colony sizes and scores of technical replicates is also produced in addition to the per-plate files.

**Table 1.** Example SGAtools output file

Row	Col	Colony size	PlateID	Query	Array	ncolony size	Score	Kvp
1	1	2943	user_dm_CAN1_12_SG-round-1.dat	CAN1	BLANK	NA	NA	(status = BG)
1	2	2899	user_dm_CAN1_12_SG-round-1.dat	CAN1	BLANK	NA	NA	(status = BG)
1	3	2585	user_dm_CAN1_12_SG-round-1.dat	CAN1	BLANK	689.333	0.436	NA
1	4	2543	user_dm_CAN1_12_SG-round-1.dat	CAN1	BLANK	742.568	0.540	NA
1	5	0	user_dm_CAN1_12_SG-round-1.dat	CAN1	YAL012W	167.455	0.031	NA
1	6	0	user_dm_CAN1_12_SG-round-1.dat	CAN1	YAL012W	163.544	0.023	NA
1	7	1452	user_dm_CAN1_12_SG-round-1.dat	CAN1	YBL003C	515.654	0.177	NA
1	8	1034	user_dm_CAN1_12_SG-round-1.dat	CAN1	YBL003C	358.912	−0.130	NA

There are nine columns in the SGAtools output. The first two columns determine the location of the colony on the plate (row and column). The third column is the raw colony size produced by image analysis. The fourth column gives the plate ID of the colony, which is usually the image file name and the .dat extension. Columns five and six give the query and array gene, if applicable, and are automatically filled with an index for array, and filename for query, if the annotation is not provided. The seventh and eighth columns give the normalized colony size and calculated interaction score. The colony size is NA if the colony fails any of the applied filters, and the reason for failure is given in the ninth column (see the table). For control genes and plates, or when the user opts not to score the colony, the score is also NA. Finally, the ninth column contains any additional information about the colony as a list of key-value pairs (kvp), separated by semicolons. For example, the first colony is filtered out, as it is surprisingly big for the plate, this is designated as ‘status = BG’. The full list of statuses is given in the help files online.

## DATA VISUALIZATION

It can be difficult to interpret results from large-scale experiments based on columns of raw data in large flat files. To help researchers get a broad overview of the screening results, SGAtools includes three visualization approaches to complement the quantitative analyses. These features can be accessed after normalization and scoring. First, the user can visualize the colony size or fitness values as a heatmap (Figure 1a), and compare it with the original image. This allows the user to verify quantification results and confirm that the assigned scores are consistent with intuition. Second, the distribution of the colony sizes or fitness can be visualized as a histogram (Figure 1a). Ranges of the histogram can be highlighted to select genes with the most extreme scores, and the selected genes are displayed below the histogram as a table. Finally, the selected genes can be tested for enrichment in Gene Ontology terms using g:Profiler (18).

The data visualization aims to maximize interactivity for the user. The raw images can be swapped with a gridded version to see the results of image analysis or hidden to optimize the viewing space for heatmaps. Hovering over individual components of the heatmap displays information about the colony (score or colony size, experiment name, array gene name). The heatmap coloring scheme can be modified to accentuate changes in the low or high ranges of the values. The selection for the histogram can also be adjusted using a draggable sliding window.

## VALIDATION

The image analysis and normalization methods re-implemented in SGAtools for medium scale use have previously been used in the context of high-throughput yeast double deletion screens [e.g. (1)]. To confirm that our implementation performs correctly, we tested its performance on simulated data, and a gold standard data set derived from high-throughput screens.

**Figure 2.**
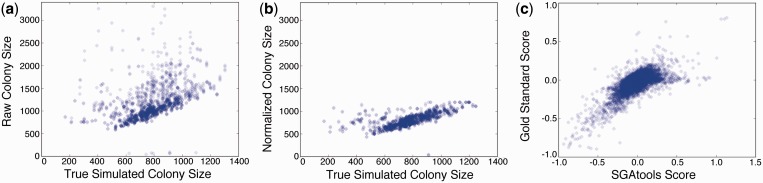
Validation of SGAtools performance. (**a** and **b**) Comparison of simulated colony sizes (*x*-axis) with ones subject to confounding experimental effects (*y*-axis) before (a, *r* = 0.42) and after (b, *r* = 0.66) SGAtools normalization. (**c**) Comparison of SGAtools interaction score (*x*-axis) with gold standard based on Costanzo *et al.* [(9), *y*-axis, *r* = 0.70].

First, we created 1000 simulated data sets from the implicit generative model, using effect sizes and variance parameters that are comparable with those of actual data. The average Pearson correlation of simulated colony sizes (not subject to systematic biases) and normalized colony sizes was 0.66, whereas the correlation of simulated colony sizes and raw unnormalized colony sizes was 0.42. The substantially stronger concordance shows that colony sizes normalized by SGAtools better capture the true underlying signal under the assumptions of this model (Figure 2a and b). Second, we verified that the obtained genetic interaction scores recapitulate data from Costanzo *et al.* (1). We selected seven genome-wide screens and scored the plates using SGAtools. The Pearson correlation of interaction scores of all combined data was 0.72 (Figure 2c), with values for the seven screens individually ranging from 0.48 to 0.90. Although the correlations are not perfect, they are as strong as those observed between biological replicates of the same screen [0.67–0.75, (13)], confirming the correct performance of the statistical model behind the SGAtools software.

>

## DISCUSSION

SGAtools is a complete simple-to-use website for analyzing small-scale array-based screens. It gives researchers without access to designated computational teams the ability to independently quantify and visualize screening results, which greatly expediates analyses. Although SGAtools was primarily developed for the yeast community with the SGA analysis in mind, it can also be useful for other types of colony size-based screens. Chemical genomic screens in yeast are already supported, and colony arrays for other microorganisms, such as *Schizosaccharomyces pombe* (24) and *Escherichia coli* (25) would be straightforward for the user to analyze.

SGAtools is aimed at quantifying relatively small-scale screens. Thus, we cannot correct for effects that can only be estimated when high-throughput data are available. For example, we have previously corrected for differences between the batch effects associated with large-scale SGA screens (13), a type of correction that becomes important when there is no precisely matched control. Also, while we flag colonies potentially affected by the competition effect (see earlier in the text), reliably correcting for it again requires large amounts of data. Finally, interaction scores are most robust when calculated using a large number of control plates, which may not available for smaller screens.

The analysis software is publicly available and can also be extended for other methodology. For example, one could opt to first score the strains, and then apply the normalization steps as recently suggested (17). Our groups are actively involved in several screening projects that use SGAtools for analysis, and we expect to continue implementing and updating any emerging best practices for the community.

## AVAILABILITY AND IMPLEMENTATION

The SGAtools website at http://sgatools.ccbr.utoronto.ca is free and open to all users, and there is no login requirement. The web application uses the Java and Scala-based Play Framework backend (http://www.playframework.org), and the Twitter Bootstrap UI library (http://twitter.github.com/bootstrap) for the frontend. Visualizations are generated using the JavaScript libraries Data Driven Documents [D3, (26)] and crossfilter (http://square.github.com/crossfilter). Image analysis is implemented using modified source code from HT colony grid analyzer (20). Normalization and scoring use the R statistical programming language (http://www.R-project.org). All the source code is available on GitHub at https://github.com/boonelab/sgatools.

## FUNDING

University of Minnesota Doctoral Dissertation Fellowship (to B.V.); National Institutes of Health grants [1R01HG005853-01, 1R01HG005084-01A1, 1R01HG005853-01 to B.V., M.C., C.L.M., B.A., C.B.]; CIHR grant [MOP-102629 to B.A., C.B.]; ORF grant [GL2-01-22 to B.A., C.B.]; NSERC Doctoral Postgraduate Scholarship (to E.K.); and Canadian Institute for Advanced Research Fellowship (to L.P.). Funding for open access charge: Personal funds, or group/institutional grants.

C*onflict of interest statement*. The funders had no role in study design, data collection and analysis, decision to publish or preparation of the manuscript.
